# Association of ECE1 gene polymorphisms and essential hypertension risk in the Northern Han Chinese: A case‒control study

**DOI:** 10.1002/mgg3.1188

**Published:** 2020-02-27

**Authors:** Hao Wang, Jielin Liu, Kuo Liu, Ya Liu, Jie Wen, Zuoguang Wang, Shaojun Wen

**Affiliations:** ^1^ Department of Hypertension Research Beijing Anzhen Hospital Capital Medical University and Beijing Institute of Heart Lung and Blood Vessel Diseases Beijing People's Republic of China

**Keywords:** endothelin converting enzyme‐1, essential hypertension, polymorphism, sex difference, the Northern Han Chinese population

## Abstract

**Background:**

The ECE1 gene polymorphisms have been studied as a candidate gene in essential hypertension, but no consensus has been reached. To systematically explore their possible association, a case**‒**control study was conducted.

**Methods:**

This study included 398 hypertensive subjects and 596 healthy volunteers as control subjects in the Northern Han Chinese. A total of 10 tag SNPs of ECE1 gene were genotyped successfully by TaqMan assay.

**Results:**

A total of 10 SNPs (rs212544, rs2076280, rs115071, rs2076283, rs9426748, rs11590928, rs212515, rs2236847, rs2282715, and rs2774028) were identified as the tag SNPs for ECE1 gene. Although no positive connection has been found in general population, several SNPs have been found to be related to EH risk in gender‐stratified subgroup analysis. In males, rs115071 T allele influenced EH risk in a protective manner, with dominant model (TT+TC vs. CC: *p* = .032, OR = 0.655, 95% CI = 0.445–0.965), additive model (TT vs. TC vs. CC: *p* = .019, OR = 0.616, 95% CI = 0.411–0.924), as well as allele comparison (T vs. C: *p* = .045, OR = 0.702, 95% CI = 0.496–0.992). While, in females, rs212544 AA genotype would increase the onset risk of EH (recessive model: AA vs. GA+GG, *p* = .024, OR = 1.847, 95% CI = 1.086–3.142). In the three haplotype blocks identified, rs2076283‐rs2236847 C‐T haplotype was associated with a decreased risk of EH (OR = 0.558, *p* = .046).

**Conclusion:**

The current case**‒**control study suggested that several SNPs and related haplotypes on ECE1 gene might be associated with the susceptibility of EH in certain gender subgroups in the Northern Han Chinese population.

## Graphical abstract

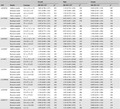

## 1 INTRODUCTION

Essential hypertension (EH) is a worldwide public health problem, which contributes greatly to the global burden of cardiovascular morbidity and mortality (Mills et al., 2016). In China, hypertension is one of the fastest escalating diseases of the past 30 years (Gao et al., 2013). According to a recent survey published on Circulation, the prevalence of hypertension was approximately 27.9% in the Chinese population over 18 years in 2012 to 2015, leading to a high prevalence of heart failure, stroke, arterial fibrillation, and multiple target organs injuries (Wang et al., 2018). EH is generally regarded as a complex disorder which results from the interplay of genetic and environmental factors. Evidences from pedigree study have suggested that the correlation between high blood pressure phenotypes is significantly stronger between parents and offspring than that between spouses, suggesting that the exploration for hypertension‐susceptibility genetic factors are also crucial for the prevention to EH besides environmental factors (Stamler, Stamler, Riedlinger, Algera, & Roberts, 1979).

Endothelin (ET) is the most potent vasoconstrictor and vasopressor up to now. It plays an important role in the occurrence and development of hypertension (Dhaun & Webb, 2019). Endothelin converting enzyme‐1 (ECE1), the key enzyme in ET system, can produce active endothelin (ET) by hydrolysis of the big endothelin (big‐ET) (Luscher & Barton, 2000). Previous studies found that some of the single‐nucleotide variants of ECE1 can influence the mRNA level of ECE1, then affect the activity of this enzyme and finally induce changes in the expression level of ET (Minamino et al., 1997; Shirai et al., 2006; Turner & Murphy, 1996).

Single‐nucleotide polymorphism (SNP) is one of the most common genetic variants in humans, and is a key factor in disease susceptibility and drug response. As far as the authors know, there are five studies studying the relationship between ECE1 SNPs and EH risk or blood pressure levels in healthy population (Banno et al., 2007; Funalot et al., 2004; Funke‐Kaiser et al., 2003; Iemitsu et al., 2006; Li et al., 2017). These studies mainly focused on three SNPs, including rs213045 (c.‐338C>A), rs213046 (c.‐839T>G) as well as rs212528 (c.616‐329A>G). Rs213045 and rs213046 locate in promoter region of ECE1 gene. Funalot found that female, elderly rs213045 AA carriers tended to have higher SBP, heart rate and average blood pressure than CA and CC genotype carriers (*p*‐value = 001, .02, and .006) (Funalot et al., 2004). Funke reported the same results after studying in female untreated EH patients. Meanwhile, his study also reported the association between rs213046 and the blood pressure level, which said that rs213046 G allele carriers have higher level of diurnal and nocturnal blood pressure (Funke‐Kaiser et al., 2003). A case**‒**control study conducted in Northern Han Chinese found that rs213045 A allele is associated with increased risk of EH (OR = 1.447, *p* = .014), however, it failed to find the relationship between rs213046 and EH risk (Li et al., 2017). Another SNP, rs212528 is in the 17^th^ intron in ECE1 gene. A case**‒**control study from Japanese population found that the EH risk of female rs212528 AA carriers increased by 0.4 folds, compared with AG and GG genotypes (Banno et al., 2007). However, AA carriers were reported to have lower level of SBP, DBP, and heart rate in another healthy Japanese population (Iemitsu et al., 2006).

It is easy to see that previous studies made great contributions, as they provided us a clue that ECE1 SNPs might be associated with EH or some other blood pressure‐related diseases. However, considering the existing studies were conducted in different populations, some in healthy participants and some in EH patients, and in different ethnics, which might be with different genetic background and influenced by different environmental factors, and most importantly they achieved inconsistent results, further studies are still in badly need. Therefore, we conducted a case**‒**control study to explore the association between the SNP loci of ECE1 gene and EH in Northern Han Chinese.

## 2 MATERIALS AND METHODS

### 2.1 Ethics statement

The current study complies with the Declaration of Helsinki. Written informed consent was obtained from all participants. The aim, design, and detailed protocol of this study were approved by the local ethics committee of Beijing Anzhen Hospital of the Capital University of Medical Sciences.

### 2.2 Study design and subjects

This is a case**‒**control study. All participants were unrelated Northern Han Chinese origin and over 18 years old. Participants in control group were recruited from the physical examination center of Anzhen Hospital affiliated to Capital University of Medical Sciences (Beijing, China) and another two examination centers at the local health stations: Liuliqiao and Guozhuang in the suburbs of Beijing. Patients in EH group were screened at hypertension clinic of Anzhen Hospital. Blood pressure was measured by trained and certified observers according to a common protocol adapted from procedures recommended by European Society of Hypertension (Mancia et al., 2013). After sitting for 30 min in a quiet room, three measurements with a standardized mercury sphygmomanometer were performed with at least 5 min intervals. One of the four bladders (standard, larger, smaller, and pediatric) was chosen and all readings were obtained from the right arm. SBP and DBP were defined according to Korotkoff I and V. Heart rate was measured by cardiac auscultation. Hypertension was defined as the average SBP ≥140 mmHg and/or the average DBP ≥90 mmHg and/or self‐reported current treatment for hypertension with antihypertensive medication. The control subjects had systolic pressure <140 mmHg and diastolic pressures <90 mmHg, respectively, and should never been diagnosed as hypertension or treated for hypertension (Mancia et al., 2013). Patients meet any of the following criteria are excluded in this study: (a) secondary hypertension, including pheochromocytoma, aldosteronism, hyperthyroidism, renal vascular disease, nephrotic syndrome, and so on; (b) white coat hypertension; (c) diabetes mellitus; (d) serious cardiovascular and cerebrovascular diseases; (e) multiple organ failure or malignant tumor, the estimated life expectancy is less than 1 year. Physical examination, a questionnaire and serum biochemical profile were administered to each of the participants. Information on smoking and drinking habits was obtained by interview. Smoker was defined as the cigarette consumer who has smoked ≥100 cigarettes, and drinker was defined as the alcohol consumer who drank ≥12 times during the year (Ge et al., 2005; Gu et al., 2006).

### 2.3 SNP identification

The tag SNP approach was used in the current study to predict the remaining common SNPs. It was conducted as follows. Firstly, the ECE1 common SNPs (minor allele frequency [MAF] >10%) were searched from the Han Chinese data sets of the International HapMap Project SNP database (http://www.hapmap.org/, HapMap Genome Browser release #27). Then, the tag SNPs with an *r* ^2^ ≥ .85 examined by Haploview 4.2 software (http://www.broad.mit.edu/mpg/haploview) were selected.

### 2.4 SNP genotyping

Blood was taken into ethylene‐diamine tetra‐acetic (EDTA)‐containing receptacles. Genomic DNA was extracted from peripheral blood according to standard phenol‐chloroform methods. SNPs were genotyped using the TaqMan assay. The ECE1 SNP Taqman probes and primers which were labeled with different fluorescent dyes were obtained from Applied Biosystems Assay‐by‐Design Service for SNP genotyping (Applied Biosystems, Foster City, CA, USA). The sample DNA was amplified by PCR following the recommendations of the manufacturer. Thermal cycling was done on a GeneAmp PCR System 9700 thermal cycler (Applied Biosystems) with the following conditions: initial denaturation and activation at 95°C for 10 min, followed by 35 cycles of 95°C for 20s, and 62°C for 1 min. Genotypes were differentiated by analyzing the fluorescence levels of PCR products using an ABI PRISM 7900HT Sequence Detector (Applied Biosystems). Genotyping was performed blindly to all other data.

### 2.5 Sample power calculation

The statistical power of this study was calculated by using Quanto software (version 1.2.4, University of Southern California). Dominant genetic model was adopted and statistical significance was set at *p* < .05. Given the available sample size, a statistical power of over 93% was achieved for detecting a risk allele with an OR of 1.5.

### 2.6 Statistical analysis

The SPSS software (version 22.0; IBM SPSS Statistics) was used for statistical analysis. Continuous variables were expressed as mean ± standard deviation. Categorical variables were shown as frequency (percentage). The independent‐samples *t* test and Pearson's χ^2^ test were used for comparisons between groups. The presence of Hardy–Weinberg equilibrium was detected by the χ^2^ test for goodness of fit based on a web program (http://ihg.gsf.de/cgi-bin/hw/hwa1.pl). Genotypic and allelic frequencies were compared between hypertensive group and control group. Binominal logistic regression was used to examine the association between ECE1 SNPs and the risk for hypertension under different models of inheritance (additive, dominant, and recessive and homozygote comparison) after adjusting for the confounding factors. Construction of the linkage disequilibrium (LD) map and haplotype blocks within ECE1 SNPs was based on genotypes and utilized Haploview software (version 4.2; http://www.broad.mit.edu/mpg/haploview/). Taking into account the covariates, haplotype‐based logistic regressive analysis was conducted with PLINK software (version 2.0, http://pngu.mgh.harvard.edu/%223Cpurcell/plink/). Haplotypes with frequencies of less than 1% were ignored in the current study. All analysis used a two‐tailed estimation of significance. The statistical significance was defined as *p* < .05.

## 3 RESULTS

### 3.1 Characteristics of the study population

Altogether 398 hypertension patients (236 males and 162 females; mean age 51.33 ± 6.88 years) and 596 healthy controls (334 males and 262 females; mean age 51.00 ± 8.71 years) were included in this study. The subjects were adequately matched for age and gender between groups. As shown in Table 1, the levels of SBP, DBP, BMI, TG, glucose, and Hcy were relatively higher in hypertension group. Levels of HDL‐C and LDL‐C were found to be lower in hypertension group, compared with controls. The similar trend was also observed in subgroups stratified by gender. The level of TCHO was lower in the total and male hypertensive patients. No significant differences were found in the following parameters between EH patients and controls in both total and gender‐stratified subgroups: age, HR, Cr, as well as incidences of smokers and drinkers.

**Table 1:** Clinical characteristic of the study population

	Overall			Male			Female		
	Hypertension （*n* = 398）	Normotension （*n* = 596）	*p*	Hypertension （*n* = 236）	Normotension （*n* = 334）	*p*	Hypertension （*n* = 162）	Normotension （*n* = 262）	*p*
Gender (Male) (*n*, %)	236 (59.3)	334 (56.0)	.309	—	—	—	—	—	—
Age (years)	51.33 ± 6.88	51.00 ± 8.71	.526	50.32 ± 6.58	50.43 ± 7.95	.855	52.80 ± 7.05	51.72 ± 9.57	.183
SBP (mmHg)^a^	135.40 ± 17.23	117.09 ± 12.02	<.001*	135.01 ± 16.99	118.17 ± 10.78	<.001*	135.95 ± 17.61	115.71 ± 13.33	<.001*
DBP (mmHg)^a^	87.33 ± 13.07	73.66 ± 9.94	<.001*	88.97 ± 12.67	76.54 ± 8.12	<.001*	84.95 ± 13.32	70 ± 10.82	<.001*
HR (bpm)	68.59 ± 9.30	69.30 ± 9.31	.249	67.79 ± 8.67	68.54 ± 9.67	.351	69.76 ± 10.07	70.26 ± 8.76	.6
BMI (Kg/m^2^)	25.01 ± 7.20	22.87 ± 7.44	<.001*	25.99 ± 6.80	24.00 ± 7.00	.001*	23.58 ± 7.54	21.82 ± 9.94	.005*
TCHO (mmol/L)	4.98 ± 1.75	5.18 ± 0.92	.033*	4.91 ± 0.99	5.17 ± 0.96	.001*	5.08 ± 2.48	5.2 ± 0.86	.497
HDL‐C (mmol/L)	1.14 ± 0.50	1.25 ± 0.36	<.001*	1.08 ± 0.26	1.17 ± 0.36	.002*	1.23 ± 0.71	1.37 ± 0.31	.006*
LDL‐C (mmol/L)	3.01 ± 0.86	3.23 ± 0.84	<.001*	3.03 ± 0.86	3.31 ± 0.86	<.001*	2.97 ± 0.85	3.13 ± 0.8	.048*
TG (mmol/L)	1.95 ± 1.61	1.55 ± 0.97	<.001*	2.11 ± 1.77	1.69 ± 1.01	.001*	1.74 ± 1.32	1.38 ± 0.88	.001*
Glu (mmol/L)	5.38 ± 0.84	5.23 ± 0.70	<.001*	5.42 ± 0.82	5.27 ± 0.54	.01*	5.39 ± 0.62	5.19 ± 0.79	.008*
Hcy (μmol/L)	9.58 ± 7.94	6.49 ± 8.24	<.001*	10.44 ± 9.14	7.46 ± 8.85	<.001*	8.32 ± 5.54	5.24 ± 7.2	<.001*
Cr (μmol/L)	73.05 ± 17.23	71.52 ± 13.81	.141	80.96 ± 15.14	80.02 ± 10.32	.408	61.45 ± 13.07	60.69 ± 9.45	.522
Smokers (*n*, %)	126 (31.7)	168 (28.2)	.24	119 (50.4)	157 (47.0)	.421	7 (4.3)	11 (4.2)	.952
Drinkers (*n*, %)	173 (43.5)	269 (45.1)	.604	156 (66.1)	231 (69.2)	.441	17 (10.5)	38 (14.5)	.232

Abbreviations: BMI, body mass index; Cr, creatinine; DBP, diastolic blood pressure; Glu, glucose; Hcy, homocysteine; HDL‐C, high‐density lipoprotein cholesterol; HR, heart rate; LDL‐C, low‐density lipoprotein cholesterol; SBP, systolic blood pressure; TCHO, total cholesterol; TG, triglyceride.

a The well‐treated patients were also included in Hypertension group.

* *p* < .05.

### 3.2 Identification and distribution of ECE1 SNPs

According to the criteria mentioned in methods, 11 tag SNPs (rs604346, rs212544, rs2076280, rs115071, rs2076283, rs9426748, rs11590928, rs212515, rs2236847, rs2282715, and rs2774028) of the ECE1 gene were selected. All these SNPs are introns on chrome 1. Rs604346 located at chr1:21256512, which is too close to rs115071 (chr1:21256510). During genotyping, we failed to design corresponding probe for it. So we excluded this loci during final SNP genotyping owing to technology limitations. The genotyping call rates for the rest 10 SNPs were all over 90%.

Distributions of genotype and allele frequencies in total and gender subgroups are shown in Table 2. All SNPs were in agreement with Hardy**‒**Weinberg equilibrium for healthy controls. In present study, no statistically significant difference was found in the genotype and allele counts between hypertension group and controls in both overall population and male subgroup. However, the genotype distribution trend of rs2076280 was different between hypertensive and normotensive ones in females. The genotype frequencies of CC, CT, and TT of rs2076280 in female hypertension patients were 66.5%, 32.9%, and 0.6%, and in control group, they were 71.6%, 24.5%, and 3.9%, respectively (*p* = .034).

**Table 2:** Distribution of allelic and genotypic frequencies of ECE1 SNPs and HWE information

	Genotype (frequency, %)			*p*	Allele (frequency, %)		*p*	HWE	
								χ^2^	*p*
rs9426748	CC	CA	AA		A	C			
Overall									
Case	142 (39.9)	165 (46.3)	49 (13.8)	—	263 (36.9)	449 (63.1)	—		
Control	240 (41.6)	260 (45.1)	77 (13.3)	.876	414 (35.9)	740 (64.1)	.643	0.246	.62
Male									
Case	83 (38.8)	100 (46.7)	31 (14.5)	—	162 (37.9)	266 (62.1)	—		
Control	138 (42.9)	145 (45)	39 (12.1)	.562	223 (34.6)	421 (65.4)	.281	0.009	.923
Female									
Case	59 (41.5)	65 (45.8)	18 (12.7)	—	101 (35.6)	183 (64.4)	—		
Control	102 (40)	115 (45.1)	38 (14.9)	.825	191 (37.5)	319 (62.5)	.597	0.357	.55
rs2774028	CC	CT	TT		T	C			
Overall									
Case	218 (55.1)	147 (37.1)	31 (7.8)	—	209 (26.4)	583 (73.6)	—		
Control	321 (54.6)	231 (39.3)	36 (6.1)	.52	303 (25.8)	873 (74.2)	.757	0.428	.513
Male									
Case	126 (53.4)	90 (38.1)	20 (8.5)	—	130 (27.5)	342 (72.5)	—		
Control	179 (53.9)	138 (41.6)	15 (4.5)	.141	168 (25.3)	496 (74.7)	.397	3.297	.069
Female									
Case	92 (57.5)	57 (35.6)	11 (6.9)	—	79 (24.7)	241 (75.3)	—		
Control	142 (55.5)	93 (36.3)	21 (8.2)	.856	135 (26.4)	377 (73.6)	.59	1.063	.303
rs212515	AA	AC	CC		C	A			
Overall									
Case	178 (44.9)	170 (42.9)	48 (12.1)	—	266 (33.6)	526 (66.4)	—		
Control	264 (44.8)	262 (44.5)	63 (10.7)	.757	388 (32.9)	790 (67.1)	.764	0.028	.867
Male									
Case	109 (46.2)	102 (43.2)	25 (10.6)	—	152 (32.2)	320 (67.8)	—		
Control	138 (41.6)	163 (49.1)	31 (9.3)	.383	225 (33.9)	439 (66.1)	.553	3.043	.081
Female									
Case	69 (43.1)	68 (42.5)	23 (14.4)	—	114 (35.6)	206 (64.4)	—		
Control	126 (49)	99 (38.5)	32 (12.5)	.496	163 (31.7)	351 (68.3)	.243	3.143	.076
rs2076283	CC	CG	GG		G	C			
Overall									
Case	110 (29.3)	190 (5.5)	76 (20.2)	—	342 (45.5)	410 (54.5)	—		
Control	162 (28)	293 (5.6)	124 (21.4)	.866	541 (46.7)	617 (53.3)	.595	0.157	.692
Male									
Case	65 (29.1)	110 (49.3)	48 (21.5)	—	206 (46.2)	240 (53.8)	—		
Control	83 (25.6)	169 (52.2)	72 (22.2)	.655	313 (48.3)	335 (51.7)	.491	0.639	.424
Female									
Case	45 (29.4)	80 (52.3)	28 (18.3)	—	136 (44.4)	170 (55.6)	—		
Control	79 (31)	124 (48.6)	52 (20.4)	.761	228 (44.7)	282 (55.3)	.942	0.069	.793
rs2236847	TT	TC	CC		C	T			
Overall									
Case	81 (20.7)	214 (54.6)	97 (24.7)	—	408 (52)	376 (48)	—		
Control	149 (25.4)	303 (51.6)	135 (23)	.232	573 (48.8)	601 (51.2)	.161	0.638	.425
Male									
Case	50 (21.4)	124 (53)	60 (25.6)	—	244 (52.1)	224 (47.9)	—		
Control	87 (26.3)	174 (52.6)	70 (21.1)	.276	314 (47.4)	348 (52.6)	.119	0.97	.325
Female									
Case	31 (19.6)	90 (57)	37 (23.4)	—	164 (51.9)	152 (48.1)	—		
Control	62 (24.2)	129 (5.4)	65 (25.4)	.392	259 (50.6)	253 (49.4)	.714	0.016	.899
rs115071	CC	CT	TT		T	C			
Overall									
Case	238 (65.7)	108 (29.8)	16 (4.4)	—	140 (19.3)	584 (80.7)	—		
Control	347 (61.3)	196 (34.6)	23 (4.1)	.316	242 (21.4)	890 (78.6)	.289	0.514	.473
Male									
Case	149 (69.3)	56 (26)	10 (4.7)	—	76 (17.7)	354 (82.3)	—		
Control	191 (60.3)	113 (35.6)	13 (4.1)	.066	139 (21.9)	495 (78.1)	.09	0.539	.463
Female									
Case	89 (60.5)	52 (35.4)	6 (4.1)	—	64 (21.8)	230 (78.2)	—		
Control	156 (62.7)	83 (33.3)	10 (4)	.914	103 (20.7)	395 (79.3)	.717	0.063	.801
rs212544	GG	GA	AA		A	G			
Overall									
Case	123 (31.4)	182 (46.4)	87 (22.2)	—	356 (45.4)	428 (54.6)	—		
Control	188 (32)	303 (51.5)	97 (16.5)	.07	497 (42.3)	679 (57.7)	.169	1.838	.175
Male									
Case	73 (31.2)	113 (48.3)	48 (20.5)	—	209 (44.7)	259 (55.3)	—		
Control	102 (30.8)	172 (52)	57 (17.2)	.556	286 (43.2)	376 (56.8)	.627	1.146	.284
Female									
Case	50 (31.6)	69 (43.7)	39 (24.7)	—	147 (46.5)	169 (53.5)	—		
Control	86 (33.5)	131 (51)	40 (15.6)	.065	211 (41.1)	303 (58.9)	.122	0.727	.394
rs11590928	CC	CT	TT		T	C			
Overall									
Case	212 (54.1)	154 (39.3)	26 (6.6)	—	206 (26.3)	578 (73.7)	—		
Control	340 (57.9)	204 (34.8)	43 (7.3)	.351	290 (24.7)	884 (75.3)	.433	2.54	.111
Male									
Case	124 (53)	96 (41)	14 (6)	—	124 (26.5)	344 (73.5)	—		
Control	196 (59.2)	115 (34.7)	20 (6)	.303	155 (23.4)	507 (76.6)	.237	0.323	.57
Female									
Case	88 (55.7)	58 (36.7)	12 (7.6)	—	82 (25.9)	234 (74.1)	—		
Control	144 (56.3)	89 (34.8)	23 (9)	.847	135 (26.4)	377 (73.6)	.894	2.804	.094
rs2076280	CC	CT	TT		T	C			
Overall									
Case	273 (69.6)	115 (29.3)	4 (1.0)	—	125 (15.9)	659 (84.1)	—		
Control	412 (70.1)	159 (27.0)	17 (2.9)	.117	193 (16.4)	983 (83.6)	.783	0.122	.727
Male									
Case	168 (71.8)	63 (26.9)	3 (1.3)	—	69 (14.7)	399 (85.3)	—		
Control	228 (68.9)	96 (29)	7 (2.1)	.633	110 (16.6)	552 (83.4)	.396	0.72	.396
Female									
Case	105 (66.5)	52 (32.9)	1 (0.6)	—	56 (17.7)	260 (82.3)	—		
Control	184 (71.6)	63 (24.5)	10 (3.9)	.034*	83 (16.1)	431 (83.9)	.555	2.309	.129
rs2282715	GG	GA	AA		A	G			
Overall									
Case	109 (27.8)	191 (48.7)	92 (23.5)	—	375 (47.8)	409 (52.2)	—		
Control	177 (30.1)	277 (47.1)	134 (22.8)	.741	545 (46.3)	631 (53.7)	.518	1.637	.201
Male									
Case	61 (26.1)	112 (47.9)	61 (26.1)	—	234 (50)	234 (50)	—		
Control	102 (30.8)	160 (48.3)	69 (20.8)	.259	298 (45)	364 (55)	.098	0.183	.669
Female									
Case	48 (30.4)	79 (50)	31 (19.6)	—	141 (44.6)	175 (55.4)	—		
Control	75 (29.2)	117 (45.5)	65 (25.3)	.402	247 (48.1)	267 (51.9)	.336	1.995	.158

* *p* < .05.

### 3.3 Single SNP association analysis

Binominal logistic regression analysis was performed after adjusting for confounding risk variables, including gender, age, BMI, TCHO, HDL‐C, LDL‐C, TG, glucose, and Hcy, with the summary of results listed in Table 3. No positive association between the 10 ECE1 SNPs and EH risk could be found in any genetic models.

**Table 3:** Association of ECE1 polymorphisms with EH under different genetic models after adjustment for confounding factors

SNP	Models	Genotype	Overall		Male		Female	
			OR (95% CI)^a^	*p* a	OR (95% CI)^b^	*p* b	OR (95% CI)^c^	*p* c
rs9426748	Additive models	AA vs. CA vs. CC	1.063 (0.786–1.437)	.691	1.116 (0.754–1.652)	.584	0.942 (0.585–1.519)	.807
	Dominant model	CA+AA vs. CC	1.071 (0.806–1.422)	.637	1.165 (0.805–1.685)	.419	0.906 (0.578–1.420)	.666
	Recessive model	AA vs. CA+CC	1.050 (0.702–1.571)	.813	1.253 (0.739–2.124)	.402	0.824 (0.435–1.562)	.553
	Allele comparison	A vs. C	1.137 (0.916–1.412)	.245	1.206 (0.912–1.595)	.189	0.999 (0.704–1.417)	.995
rs2774028	Additive models	TT vs. CT vs. CC	0.923 (0.692–1.230)	.584	0.921 (0.637–1. 333)	.663	0.958 (0.606–1.515)	.855
	Dominant model	CT+TT vs. CC	0.963 (0.732–1.267)	.789	0.995 (0.697–1.420)	.978	0.930 (0.603–1.435)	.743
	Recessive model	TT vs. CT+CC	1.292 (0.754–2.213)	.352	1.874 (0.887–3.959)	.1	0.813 (0.360–1.836)	.618
	Allele comparison	T vs. C	0.977 (0.774–1.234)	.847	0.997 (0.739–1.346)	.986	0.949 (0.653–1.378)	.783
rs212515	Additive models	CC vs. CA vs. AA	0.967 (0.723–1.293)	.82	0.825 (0.569–1.196)	.309	1.208 (0.760–1.920)	.424
	Dominant model	CA+CC vs. AA	1.020 (0.775–1.342)	.889	0.875 (0.614–1.249)	.463	1.234 (0.803–1.898)	.338
	Recessive model	CC vs. CA+AA	1.283 (0.835–1.973)	.256	1.305 (0.716–2.376)	.385	1.206 (0.645–2.255)	.558
	Allele comparison	C vs. A	1.074 (0.864–1.334)	.521	1.016 (0.767–1.347)	.911	1.144 (0.809–1.618)	.446
rs2076283	Additive models	GG vs. CG vs. CC	0.974 (0.702–1.352)	.875	0.833 (0.542–1.280)	.404	1.221 (0.726–2.055)	.452
	Dominant model	CG+GG vs. CC	0.952 (0.698–1.298)	.755	0.847 (0.565–1.270)	.422	1.124 (0.686–1.844)	.642
	Recessive model	GG vs. CG+CC	0.923 (0.655–1.301)	.648	1.005 (0.651–1.552)	.982	0.790 (0.442–1.414)	.428
	Allele comparison	G vs. C	1.011 (0.821–1.246)	.915	0.986 (0.754–1.289)	.918	1.061 (0.752–1.498)	.735
rs2236847	Additive models	CC vs. CT vs. TT	1.361 (0.965–1.920)	.079	1.177 (0.759–1.824)	.467	1.841 (1.052–3.222)	.033*
	Dominant model	CT+CC vs. TT	1.370 (0.986–1.902)	.06	1.256 (0.827–1.907)	.284	1.681 (0.984–2.872)	.057
	Recessive model	CC vs. CT+TT	1.119 (0.812–1.543)	.492	1.308 (0.861–1.986)	.208	0.844 (0.508–1.403)	.514
	Allele comparison	C vs. T	1.116 (0.910–1.370)	.292	1.139 (0.875–1.481)	.333	1.062 (0.763–1.476)	.722
rs115071	Additive models	TT vs. CT vs. CC	0.766 (0.565–1.040)	.088	0.616 (0.411–(0.924)	.019*	1.060 (0.663–1.693)	.809
	Dominant model	CT+TT vs. CC	0.790 (0.590–1.060)	.116	0.655 (0.445–(0.965)	.032*	1.067 (0.678–1.680)	.778
	Recessive model	TT vs. CT+CC	1.142 (0.571–2.283)	.707	1.175 (0.485–2.848)	.721	1.115 (0.363–3.422)	.849
	Allele comparison	T vs. C	0.817 (0.628–1.062)	.13	0.702 (0.496–(0.992)	.045*	1.050 (0.697–1.582)	.814
rs212544	Additive models	AA vs. GA vs. GG	0.984 (0.721–1.343)	.919	1.012 (0.675–1.517)	.954	0.924 (0.568–1.504)	.751
	Dominant model	GA+AA vs. GG	1.063 (0.792–1.427)	.685	1.026 (0.699–1.504)	.896	1.107 (0.700–1.750)	.665
	Recessive model	AA vs. GA+GG	1.335 (0.942–1.892)	.105	1.060 (0.662–1.696)	.809	1.847 (1.086–3.142)	.024*
	Allele comparison	A vs. G	1.121 (0.912–1.378)	.277	0.996 (0.763–1.301)	.978	1.353 (0.975–1.877)	.071
rs11590928	Additive models	TT vs. CT vs. CC	1.159 (0.867–1.548)	.319	1.399 (0.964–2.029)	.077	0.947 (0.595–1.507)	.82
	Dominant model	CT+TT vs. CC	1.135 (0.862–1.494)	.368	1.357 (0.950–1.938)	.093	0.932 (0.604–1.438)	.75
	Recessive model	TT vs. CT+CC	0.948 (0.554–1.622)	.844	0.978 (0.464–2.061)	.953	0.898 (0.412–1.959)	.787
	Allele comparison	T vs. C	1.114 (0.882–1.408)	.365	1.211 (0.897–1.637)	.212	0.997 (0.685–1.451)	.986
rs2076280	Additive models	TT vs. CT vs. CC	1.172 (0.866–1.587)	.305	1.011 (0.680–1.501)	.959	1.381 (0.865–2.204)	.176
	Dominant model	CT+TT vs. CC	1.120 (0.833–1.506)	.454	1.003 (0.680–1.479)	.988	1.257 (0.795–1.990)	.328
	Recessive model	TT vs. CT+CC	0.532 (0.188–1.511)	.236	0.870 (0.203–3.724)	.851	0.358 (0.074–1.735)	.202
	Allele comparison	T vs. C	1.092 (0.827–1.441)	.535	1.005 (0.697–1.450)	.979	1.205 (0.780–1.861)	.401
rs2282715	Additive models	AA vs. GA vs. GG	0.948 (0.686–1.308)	.744	1.040 (0.679–1.591)	.858	0.865 (0.525–1.426)	.57
	Dominant model	GA+AA vs. GG	0.984 (0.728–1.330)	.915	1.147 (0.770–1.709)	.5	0.822 (0.515–1.312)	.411
	Recessive model	AA vs. GA+GG	1.102 (0.797–1.522)	.558	1.369 (0.902–2.075)	.14	0.808 (0.482–1.354)	.418
	Allele comparison	A vs. G	1.039 (0.847–1.275)	.714	1.161 (0.892–1.510)	.268	0.888 (0.639–1.233)	.477

Abbreviations: CI, confidence interval; OR, odds ratio; SNP, single‐nucleotide polymorphism.

a OR adjusted for gender, age, BMI, TCHO, HDL‐C, LDL‐C, TG, glucose, and Hcy.

b OR adjusted for age, BMI, TCHO, HDL‐C, LDL‐C, TG, glucose, and Hcy.

c OR adjusted for age, BMI, TCHO, HDL‐C, TG, glucose, and Hcy.

* *p* < .05.

However, in the further analysis stratified by gender, it was found that rs115071 was significantly associated with EH risk in males under both dominant model (TT+TC vs. CC: *p* = .032, OR = 0.655, 95% CI = 0.445–0.965) and additive model (TT vs. TC vs. CC: *p* = .019, OR = 0.616, 95% CI = 0.411–0.924), as well as also in allele comparison (T vs. C: *p* = .045, OR = 0.702, 95% CI = 0.496–0.992), which suggested that male T allele carriers of rs115071 have lower risk for EH. In females, rs212544 was found to be associated with EH under recessive model (AA vs. GA+GG: *p* = .024, OR = 1.847, 95% CI = 1.086–3.142), which indicated that female AA carriers might be more susceptible to EH. Although significant association was also observed between rs2236847 and EH in females under addictive model (CC vs. CT vs. TT: *p* = .033, OR = 1.841, 95% CI = 1.052–3.222), no positive result was found in other genetic models for this SNP.

For the other SNPs including rs604346, rs2076280, rs2076283, rs9426748, rs11590928, rs212515, rs2282715, and rs2774028, no significant result has been found.

### 3.4 Haplotype association analysis

**Figure 1:**
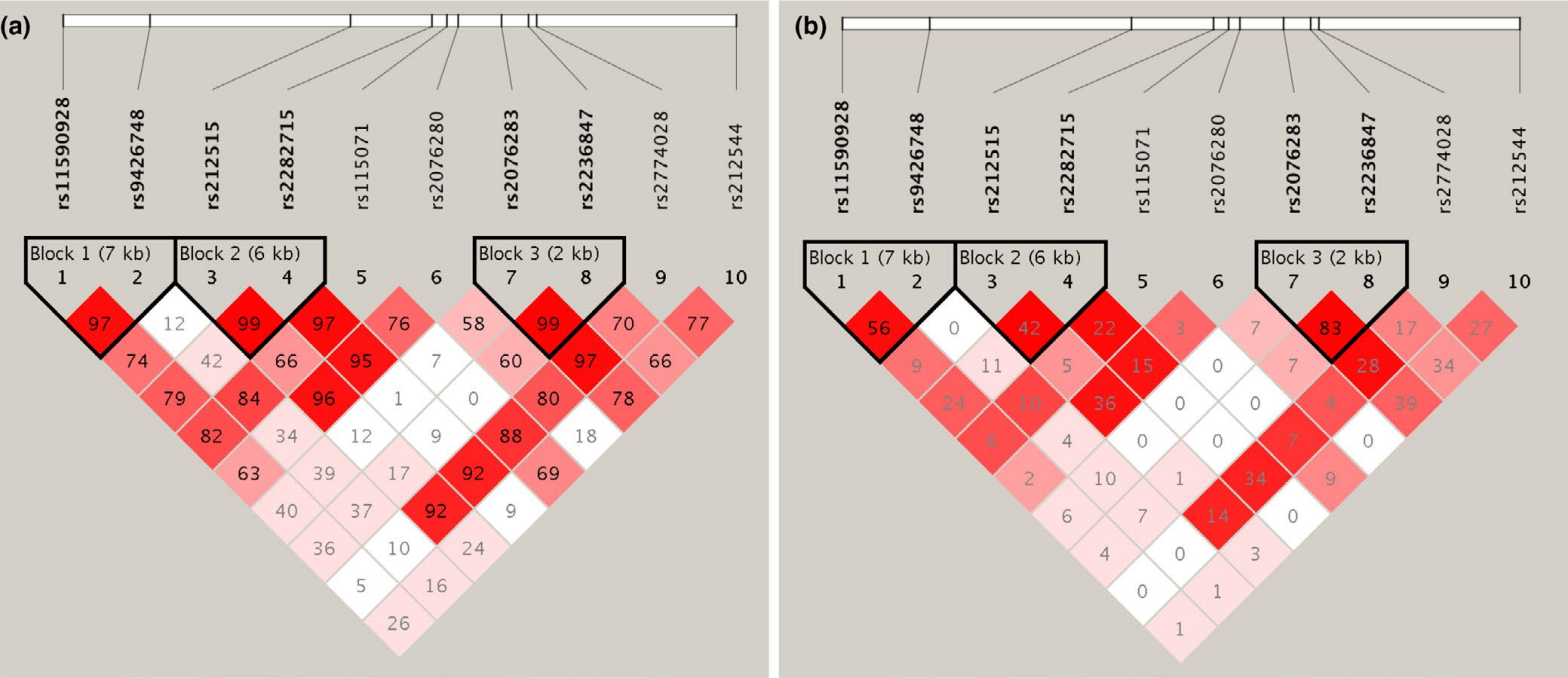
Linkage disequilibrium (LD) blocks defined by the Haploview program based on the solid spine of LD method. A. represents LD results of D’; B. represents LD results of *r* ^2^

As Figure 1 revealed, three LD blocks (rs11590928‐rs9426748, rs212515‐rs2282715, and rs2076283‐rs2236847) were identified using the standard definition of D^’^ and *r* ^2^. After adjustment for the confounding factors including gender, age, TG, TCHO, LDL‐C, HDL‐C, glucose, and Hcy with the PLINK software, we found that the haplotype C‐T (rs2076283‐rs2236847) carriers have lower risk for EH (OR = 0.558, *p* = .046) (Table 4).

**Table 4:** Haplotype analysis of ECE1 polymorphisms in case and control groups

Block	SNP1	SNP2	Haplotype	Frequency		OR^a^	*p* a	OR^b^	*p* b
				Case	Control				
1	rs11590928	rs9426748	T‐A	0.254	0.245	1.09	.43	1.09	.474
			C‐A	0.111	0.114	0.96	.793	0.94	.709
			C‐C	0.626	0.638	0.92	.398	0.929	.484
2	rs212515	rs2282715	A‐A	0.477	0.462	1.06	.539	1.03	.751
			C‐G	0.33	0.328	1.01	.927	1.06	.562
			A‐G	0.192	0.209	0.899	.36	0.871	.27
3	rs2076283	rs2236847	G‐T	0.446	0.463	0.955	.625	0.936	.515
			C‐T	0.033	0.048	0.688	.132	0.588	.046*
			C‐C	0.519	0.486	1.12	.242	1.18	.115

Abbreviations: CI, confidence interval; OR, odds ratio; SNP, single‐nucleotide polymorphism.

a ORs and *p*‐values for the haplotype‐based association analysis with a specific haplotype compared with the others.

b ORs and *p*‐values for the haplotype‐based logistic regression analysis after adjusting for gender, age, BMI, TCHO, HDL‐C, LDL‐C, TG, glucose, and Hcy.

* *p* < .05.

## 4 DISCUSSION

In the present population, we conducted a case**‒**control study on the association between the ECE1 polymorphisms and the susceptibility of individuals to EH. A total of 11 SNPs were identified as the tag SNPs for ECE1 gene. Although no positive connection has been found in general population, several SNPs have been found to be related to EH risk in gender‐stratified subgroup analysis. In males, rs115071 T allele influenced EH risk in a protective manner. While, in females rs212544 AA genotype would increase the onset risk of EH. In the three haplotype blocks, rs2076283‐rs2236847 C‐T haplotype was associated with decreased risk of EH.

Previous studies also found the possible relationship between ECE1 SNPs and EH risk, but as no common SNP has been included in current study and them, we could not make further comparison. But, based on the following reasons, the authors believe that the results obtained in the current paper are rational. First, compared with earlier studies, the sample size of the present study was relatively larger. It maintained adequate statistical power, at the same time, evaded the possibility of overfitting in multivariate regression. Second, as the Han Chinese population was considered be intricately substructured, corresponding roughly to Northern Han, Central Han, and Southern Han, based on the populations of the geographical origins (Wen et al., 2004; Xu et al., 2009), we confined our study population in Northern Han Chinese, to minimize both the racial differences and the influences induced by factors such as environmental factors, economic level, geographical location, climate, eating habits, and lifestyle. In addition, though all the 10 tag SNPs identified by Haplotype software were in the intron region of ECE1 gene, it is not surprising, as most tag SNPs detected are in intron fragment for the considerable proportion of introns on gene (Sakharkar, Chow, & Kangueane, 2004). Introns could not be translated into amino acid directly, but they play critical roles in the regulation of gene expression (Jo & Choi, 2015; Le Hir, Nott, & Moore, 2003). Besides, most tag SNPs are in close linkage disequilibrium with other functional polymorphisms (Dong et al., 2017; Grossi et al., 2018). For this purpose, we made a further confirmation by using the Haploreg v4 (http://pubs.broadinstitute.org/mammals/haploreg/haploreg_v4.php) to predict the potential function of the two positive polymorphisms, rs115071 and rs212544 (Ward, & Kellis, 2012). The results showed that there were 3 and 12 SNPs, respectively, being in linkage disequilibrium (LD) with the two SNPs mentioned above (*r* ^2^ ≥ .8). These 15 SNPs were associated with promoter and enhancer histone marks, DNAse, proteins bound, motifs changed (e.g., TATA‐binding protein, NF‐κB, E2F, Pou2f2, and so on) and selected eQTL hits, many of which were proved to be involved with blood pressure regulation (Lazar et al., 2006; Li et al., 2019; Prisco et al., 2014). Besides of inner authenticity, all the participants included in current study are unrelated, and all candidate SNPs satisfied HWE, so the results also have good external objective reality within certain scale.

The gender‐specific associations found here are similar to the findings of previous studies (Banno et al., 2007; Funalot et al., 2004; Funke‐Kaiser et al., 2003; Song, Ma, Wang, Chen, & Zhong, 2019). In clinic, Tostes found that the level of ET‐1 in male patients with hypertension was significantly higher than that in females (Tostes et al., 2008). This difference also existed in animals (Intapad, Ojeda, Varney, Royals, & Alexander, 2015). Some scholars tried to make explanations from the perspective of sex hormone. For example, Polderman found that male hormones could increase the level of plasma ET expression, while estrogen would reduce the level of plasma ET. And he believed that sex hormones might change the regulatory activity of ET on kidney water and salt metabolism (Polderman et al., 1993). Other studies have shown that, because ECE1 is expressed not only in endothelial cells, but also in steroid‐producing cells in the corpus luteum of the female ovary, the expression level of ECE1 would be affected by the menstrual cycle (Dubey, Jackson, Keller, Imthurn, & Rosselli, 2001; Levy et al., 2001; Webb, Ghatei, McNeill, & Collins, 2000). All of these factors may counteract or enhance the role of gene mutation. But reasons above did not account for the whole problem, because in Funalot's study, all subjects were postmenopausal women, and only 11.5% of the population received hormone replacement therapy (Funalot et al., 2004). Therefore, this issue still needs to be further explored in the future (Gohar & Pollock, 2018).

Although we tried our best to give consideration to both inner authenticity and the representativeness of the sampling population, however, for financial constraints, there were still some limitations in current study. First is the potential for population groups. It is inevitable that participants in control group might develop EH later in life. However, the subjects were adequately matched for age and gender between groups. As we can see, the mean age was comparable in the two sets. And most patients have passed the high‐risk age of EH. Second, it is noticeable that rs2236847 was also related to EH in females under addictive model, but no positive result was found in other genetic models for this SNP. Besides, the positive haplotype of rs2076283‐rs2236847 identified in current study was originated from two negative SNPs when working alone. So results achieved in this study need further exploration. Third, only common tag SNPs with the MAF over 10% were analyzed here, other functional SNPs with lower frequency are still worthy of study.

Finally in a word, the current study suggested that several SNPs and related haplotypes on ECE1 gene might be associated with the susceptibility of EH, which providing evidences to the early prevention, risk assessment and individualized therapy of EH to some extent. Due to the mentioned limitations, further researches are needed to confirm the above deduction.

## 5 DATA AVAILABLE STATEMENT

The data that support the findings of this study are available from the corresponding author upon reasonable request.

## CONFLICT OF INTEREST

The authors report no conflict of interest.
